# Comparison of the cytotoxic effects of *Juniperus sabina* and *Zataria multiflora* extracts with *Taxus baccata* extract and Cisplatin on normal and cancer cell lines

**DOI:** 10.4103/0973-1296.62894

**Published:** 2010-05-05

**Authors:** M. Shokrzadeh, M. Azadbakht, N. Ahangar, H. Naderi, S. S. Saeedi Saravi

**Affiliations:** *Department of Toxicology-Pharmacology, Faculty of Pharmacy, Mazandaran University of Medical Sciences, Sari, Mazandaran Pharmaceutical Sciences Research Center, Iran*; 1 *Department of Pharmacognosy, Faculty of Pharmacy, Mazandaran University of Medical Sciences, Sari, Mazandaran Pharmaceutical Sciences Research Center, Iran*

**Keywords:** Cell line, colonogenic assay, cytotoxicity, *Juniperus sabina*, *Zataria multiflora*

## Abstract

Isolation and identification of some potent anti-tumor compounds from medicinal plants has motivated researchers to screen different parts of plant species for the determination of anti-tumor effects. In this study, cytotoxic effects and IC_50_ of specific concentrations of hydro-alcoholic extracts of fruits of *Juniperus sabina* and leaves of *Zataria multiflora* were compared with hydro-alcoholic extract of bark of *Taxus baccata* and Cisplatin, well-known anticancer compounds, on normal (CHO and rat fibroblast) and cancer (HepG2 and SKOV3) cell lines. The hydro-alcoholic extracts of the plants were prepared by percolation. The cytotoxic effects and IC_50_ of the extracts on the cell lines were studied followed by colonogenic assay after 72 h incubation. The results showed that the extract of *Juniperus sabina* possesses lower IC_50_ in comparison with *Zataria multiflora* extract on all 4 normal and cancer cell lines (*P*<0.05); but, IC_50_ of the *Juniperus sabina* extract was significantly higher than the *Taxus baccata* extract and Cisplatin on all 4 normal and cancer cell lines (*P*<0.05). As a result, it is concluded that the extract of *J. sabina* has almost similar cytotoxicity with the extract of *Taxus baccata* on cancer cells.

## INTRODUCTION

Isolation and identification of some potent anti-tumor compounds, such as colchicine, Vinca alkaloids, and also docetaxol and paclitaxel (taxol), as one of the most consumed natural anticancer compounds have encouraged scientists to study the toxico-pharmacological effects of different parts of plant species against cancer cell lines.[1–8]

It has been previously reported that all parts of the yew plant contain poisonous taxine alkaloids. Taxines save their toxic effects during the year,[9] with its maximal concentration during winter.[10] However, previous investigations revealed that different parts of some species of Iranian *Juniperus sabina* possess cytotoxic effects on some human cancer cell lines.[11] The potent compound of the species of this plant is podophyllotoxin, but active ingredients of other species are lignan, silicicolin called desoxypodophyllotoxin. Further investigations on the leaves of several genera of *Juniperus sabina* (Taxus, P., Libocedrus, Podocarpus, Chamacyparis, and Callitris) showed the presence of cytotoxic compounds or tumor necrotizing substances.[1–2] Taxol is an intense anti-tumor compound extracted from *Taxus* species. However, difficulty of obtaining this compound from yew trees has limited its clinical use.[12]

*Zataria multiflora Boiss* belongs to the family Laminaceae that geographically grows in Iran, Pakistan and Afqanistan.[13,14] This plant (vernacular name of Avishan Shirazi, in Iran) has been traditionally used as an antiseptic, anesthetic and anti-spasmodic drug.[14] Also, this plant is extensively used as a flavor ingredient in Iranian food. The main constituents of its essential oil are phenolic compounds such as carvacrol and thymol.[15] In a study, Basti, Misaghi and Khaschabi reported that essential oil of *Z. multiflora* showed inhibitory effects on *Salmonella typhimurium* and *Staphylococcus aureus* in brain heart infusion (BHI) broth medium.[16]

Cisplatin (cis-dichlorodiammineplatinum-II) gained a widespread use against various malignant tumors in experimental animals[17,18] and various human malignancies.[19] Most of the biological effects of Cisplatin have been well documented[20,21] with numerous reports indicating that the cellular DNA could be the primary target in exposure to Cisplatin.[22,23] However, the therapeutic efficacy of Cisplatin is limited due to cellular drug resistance[24] and its side effects, such as delayed nausea, vomiting and nephrotoxicity.[25] Also, an increased risk of development of secondary malignancies in animals/patients treated with Cisplatin has also been reported.[26,27] To develop therapeutic effects and diminish the side effects, new analogs of Cisplatin are produced[28] and combination therapy of Cisplatin and its analogs has been tried.[29]

In this investigation, cytotoxic effects and IC_50_ of specific concentrations of hydro-alcoholic extracts of fruits of *Juniperus sabina* and leaves of *Zataria multiflora* were compared with hydro-alcoholic extract of bark of *Taxus baccata* and Cisplatin, as well as known herbal and chemical anticancer compounds on normal (CHO and rat fibroblast) and cancer (HepG2 and SKOV3) cell lines.

## MATERIALS AND METHODS

### Plant material

Fruits of *J. Sabina* and bark of *T. baccata* were collected from the northern regions of the Iran (Galugah and Neka in Mazandaran province) in September 2007. Also, leaves of *Z. multiflora* were collected from downtown of Shiraz (Fars province). The plant specimen was identified by the Department of Pharmacognosy, Sari faculty of Pharmacy, and stored at −20°C.

### Extraction and isolation

A measured quantity of 50 g of dried and powdered parts of each plant was chopped and soaked in 75 ml of ethanol (80% v/v) for 24 h and then percolated (5 h, 30 drops/min).[18] The extracts were separately concentrated by rotary evaporator, dried in oven at 45°C and dissolved in 500 ml of filtered and sterilized water (using 0.22 μm microbiological filters) containing 0.1% ethanol. The specific concentrations of the hydro-alcoholic extracts (5, 25, 50, 100 and 150 μg/ml) were prepared using phosphate buffer (pH=7.4).

### Cell lines

CHO (Normal human ovarian cells), normal rat fibroblast, HepG2 (Human hepatocarcinoma) and SKOV3 (Human ovary carcinoma) cell lines were purchased from Pasture Institute (Tehran, Iran).

The completed media were sterilized by 0.22 μ microbiological filters and kept at 4°C before use.

### Colonogenic assay

In Colonogenic assay, 50 μl of DMEM/F12 including 500-700 cells were added to 3 wells of 6well/plates for each concentration of the extracts and Cisplatin. Then, they were incubated for 48 h. After incubation, the cell lines were exposed to 50 μl of 0 (phosphate buffer), 5, 25, 50, 100, 150 μg/ml of hydro-alcoholic extracts of *Juniperus Sabina, Zataria multiflora* and *Taxus baccata*, and 50 μl of 0, 2.5, 5, 10, 25 μg/ml of Cisplatin for 2 h; and then washed using sterile normal saline 0.09%.

Then, 4 ml of fresh culture media was added to the wells, and incubated for seven days. After this period, the contents of wells were excluded; the cells were fixed by formalin 9%, and dyed by trypan blue 4% (w/v) for 20 min. Then, trypan blue was excluded, and the six well/plates were washed using sterile normal saline 0.09%. At the end, the dyed colonies were counted by light microscope.

### Statistical analysis

Prism ver.3 Software was used to perform statistical analysis. One-way ANOVA method followed by Tukey test was used to determine the differences among the groups (*P*<0.05).

## RESULTS

The results showed that the extract of *Juniperus sabina* possesses lower IC_50_ in comparison with the *Zataria multiflora* extract on all 4 normal and cancer cell lines (*P*<0.05). But, IC_50_ of the *Juniperus sabina* extract was significantly higher than the extract of *Taxus baccata* and Cisplatin on all 4 normal and cancer cell lines (*P*<0.05) [Table 1]. The lower IC_50_ represent the higher potency of a compound to inhibit the growth of cells and cause toxicity and death of cells.

**Table 1:** The evaluated IC_50_ of the *Juniperus sabina, Zataria multiflora* and Taxus baccata extracts, and Cisplatin on the selected normal and cancer cell lines.

Cell lines compound	Cancer cell line		Normal cell line	
		
	HepG2 (1C_50_*±SD)**	SKOV3 (1C_50_*±SD)**	CHO (1C_50_*±SD)**	Fibroblast (1C_50_*±SD)**
*Zataria multiflora* (Top Flower)	2.2±32.3	0.4±29.6	39.5±1.4	40±2.2
*Juniperus sabina* (Fruit)	1.2±25.9	0.8±21.7	29.2±1.8	27.3±2.1
*Taxus baccata* (Bark)	0.8±16.6	0.7±12.2	20.4±1.2	19.1±1.1
*Cisplatin*	0.07±0.87	0.08±0.99	5.5±0.21	1.6±0.21

* μg/ml;

** *P*< 0.05

**Figure 1:**
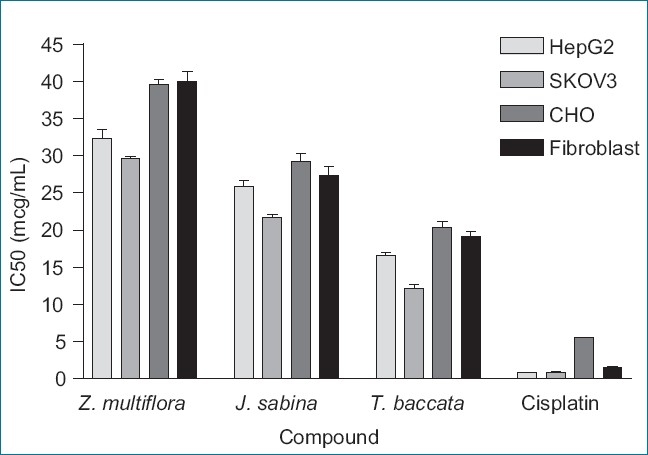
Comparison of the IC_50_ of the *Juniperus sabina* and *Zataria multiflora* extracts with the *Taxus baccata* extract and Cisplatin on the selected normal and cancer cell lines

Comparison of the evaluated IC_50_ of the *Juniperus Sabina* and *Zataria multiflora* extracts with the *Taxus baccata* extract and Cisplatin on normal and cancer cell lines are showed at Figure 1. However, the lowest and highest IC_50_ was related to cisplatin and hydro-alcoholic extract of *Zataria multiflora* in all cell lines. IC_50_ of the compounds on the 4 cell lines increased according to the rank order of cells Cisplatin < *Taxus baccata* < *Juniperus sabina* < *Zataria multiflora*.

On the other hand, the highest and lowest cytotoxicity of Cisplatin was related to HepG2 (IC_50_ = 0.87±0.07 μg/ml) and CHO cell lines (IC_50_ = 5.5±0.21 μg/ml).

The IC_50_ of cisplatin on the 4 cell lines increased according to the rank order of cells CHO > Fibroblast > SKOV3 > HepG2.

On the other hand, the IC_50_ of hydro-alcoholic extract of *Taxus baccata* on the 4 cell lines increased according to the rank order of cells CHO > Fibroblast > HepG2 > SKOV3.

Also, the IC_50_ of hydro-alcoholic extract of *Juniperus sabina* on the 4 cell lines decreased according to the rank order of cells CHO < Fibroblast < HepG2 < SKOV3.

The IC_50_ of hydro-alcoholic extract of *Zataria multiflora* on the 4 cell lines decreased according to the rank order of cells Fibroblast < CHO < HepG2 < SKOV3.

## DISCUSSION

According to the results, IC_50_ of the *Juniperus sabina* and *Zataria multiflora* extracts, and Cisplatin, as drug control positive compound and *Taxus baccata* extract, as plant control positive on normal cell lines were higher than that on cancer cell lines. This difference can be resulted from dysfunction of cellular organisms following cancer incidence which cause higher rate of proliferation and increased cellular intake. Also, defensive disorders and effusion insufficiency to escape toxic substances from cells can lead to lower necessity to amounts of cytotoxic compounds to inhibit the growth of cancer cells in comparison with normal cells.[6,8]

Determination of viability percent of the cells treated with the extract of *Juniperus sabina* and its insignificant differences with IC_50_ of the extract of *Taxus baccata*, as a common natural anticancer product, allows us to conclude that extracts of different parts of *Juniperus sabina* are good candidates for further studies of activity-monitored fractionation to identify their active components.

## CONCLUSION

In this study, we have determined the cytotoxic activity of hydroalcoholic extracts of *Juniperus Sabina* and *Zataria multiflora* on cancer cell lines and compared their IC_50_ with the hydroalcoholic extract of *Taxus baccata* and Cisplatin. We have observed that *Juniperus sabina* has insignificant differences with IC_50_ of the extract of *Taxus baccata* on HepG2 and SKOV3 cancer cells. This result presented another novel approach for the treatment ofsome cancers.
